# Targeting triple-negative breast cancer cells with the histone deacetylase inhibitor panobinostat

**DOI:** 10.1186/bcr3192

**Published:** 2012-05-21

**Authors:** Chandra R Tate, Lyndsay V Rhodes, H Chris Segar, Jennifer L Driver, F Nell Pounder, Matthew E Burow, Bridgette M Collins-Burow

**Affiliations:** 1 Department of Medicine, Section of Hematology and Medical Oncology, Tulane University Health Sciences Center, 1430 Tulane Ave, New Orleans, LA 70112, USA

**Keywords:** Panobinostat, LBH589, triple-negative breast cancer, xenograft, histone deacetylase inhibitor, E-cadherin, CDH1, epithelial-to-mesenchymal transition

## Abstract

**Introduction:**

Of the more than one million global cases of breast cancer diagnosed each year, approximately fifteen percent are characterized as triple-negative, lacking the estrogen, progesterone, and Her2/neu receptors. Lack of effective therapies, younger age at onset, and early metastatic spread have contributed to the poor prognoses and outcomes associated with these malignancies. Here, we investigate the ability of the histone deacetylase inhibitor panobinostat (LBH589) to selectively target triple-negative breast cancer (TNBC) cell proliferation and survival *in vitro *and tumorigenesis *in vivo*.

**Methods:**

TNBC cell lines MDA-MB-157, MDA-MB-231, MDA-MB-468, and BT-549 were treated with nanomolar (nM) quantities of panobinostat. Relevant histone acetylation was verified by flow cytometry and immunofluorescent imaging. Assays for trypan blue viability, 3-(4,5-dimethylthiazol-2-yl)-2,5-diphenyltetrazolium bromide (MTT) proliferation, and DNA fragmentation were used to evaluate overall cellular toxicity. Changes in cell cycle progression were assessed with propidium iodide flow cytometry. Additionally, qPCR arrays were used to probe MDA-MB-231 cells for panobinostat-induced changes in cancer biomarkers and signaling pathways. Orthotopic MDA-MB-231 and BT-549 mouse xenograft models were used to assess the effects of panobinostat on tumorigenesis. Lastly, flow cytometry, ELISA, and immunohistochemical staining were applied to detect changes in cadherin-1, E-cadherin (CDH1) protein expression and the results paired with confocal microscopy in order to examine changes in cell morphology.

**Results:**

Panobinostat treatment increased histone acetylation, decreased cell proliferation and survival, and blocked cell cycle progression at G2/M with a concurrent decrease in S phase in all TNBC cell lines. Treatment also resulted in apoptosis induction at 24 hours in all lines except the MDA-MB-468 cell line. MDA-MB-231 and BT-549 tumor formation was significantly inhibited by panobinostat (10 mg/kg/day) in mice. Additionally, panobinostat up-regulated CDH1 protein *in vitro *and *in vivo *and induced cell morphology changes in MDA-MB-231 cells consistent with reversal of the mesenchymal phenotype.

**Conclusions:**

This study revealed that panobinostat is overtly toxic to TNBC cells *in vitro *and decreases tumorigenesis *in vivo*. Additionally, treatment up-regulated anti-proliferative, tumor suppressor, and epithelial marker genes in MDA-MB-231 cells and initiated a partial reversal of the epithelial-to-mesenchymal transition. Our results demonstrate a potential therapeutic role of panobinostat in targeting aggressive triple-negative breast cancer cell types.

## Introduction

Over 200,000 new cases of invasive breast cancer are diagnosed in the United States each year and approximately 40,000 of the patients diagnosed will die from the disease [1]. Breast cancers are routinely classified by stage, pathology, grade and expression of estrogen receptor (ER), progesterone receptor (PR) or human epidermal growth factor receptor (Her2/neu). Current successful therapies include hormone-based agents that directly target these receptors [2,3]. Triple-negative breast cancer (TNBC) is a heterogeneous subset of neoplasms that is defined by the absence of these targets [4-6]. Approximately 15% of globally diagnosed breast cancers are designated as ER-, PR- and Her2/neu-negative [1,7,8]. Studies have shown that tumors of this aggressive subtype are of higher histological grade, affect a disproportionate number of young women, and are more likely to recur earlier at distant sites, resulting in poor overall prognoses [4,5,9,10]. To improve outcomes of TNBC, we must unravel its biological pathways and modes of progression and use that knowledge to develop novel targets and therapies.

Histone deacetylase inhibitors (HDACis) have emerged as a promising new class of multifunctional anticancer agents [11,12]. That promise lies in the ability of HDACis to effect multiple epigenetic changes in aberrant cells. In addition to regulating gene expression and transcription through chromatin remodeling, HDACis can also modulate a variety of cellular functions including growth, differentiation, and survival [13,14] due, in part, to their ability to enhance acetylation of a wide range of proteins, including transcription factors, molecular chaperones, and structural components [11,15,16]. Specifically, HDACis have been linked to several downstream effects in tumor cell lines which include: cell cycle arrest, induction of apoptosis, inhibition of angiogenesis, activation or inactivation of tumor suppressor genes or oncogenes, and decreased invasion and metastases [11,12,17].

Panobinostat (LBH589) is a potent pan-deacetylase inhibitor that can block multiple cancer related pathways and reverse epigenetic events implicated in cancer progression [18]. HDACs can be subdivided into two groups: zinc-dependent (Class I, II, and IV) and zinc-independent (Class III) [19]. Panobinostat is a potent inhibitor with activity against Class I, II, and IV HDAC enzymes, suggesting true pan-HDAC activity [18]. In preclinical studies, panobinostat has shown potent inhibitory activity at low nanomolar concentrations across a wide range of hematologic malignancies including lymphoma, multiple myeloma and acute myeloid leukemia [20-22]. It is also being investigated as a treatment against non-responsive solid tumors as well as tumors of the lung, thyroid, and prostate [23-26]. It has shown synergy with chemotherapeutics, radiation, demethylators, proteasome inhibitors and other agents [27-29]. Based on these preclinical findings, panobinostat and other HDACis have undergone a rapid phase of clinical development with many entering clinical trials, both as single agents or in combination with other therapies [12,23,30,31]. To date, panobinostat has demonstrated favorable clinical responses, with limited toxicity [23,32,33]. There is a critical need to develop pleiotropic therapies that specifically target the neoplasm as well as the biological pathways and markers of TNBC progression. The purpose of this study was to determine the ability of panobinostat to selectively target the TNBC subtype of breast cancer cells, assessed by its effects on the growth, survival, and tumorigenesis of a representative panel of TNBC cells. We also sought to characterize the effects panobinostat on the regulation of breast cancer genes, related signaling pathways and morphology.

## Materials and methods

### Cell lines and reagents

Human TNBC (MDA-MB-157, MDA-MB-231, MDA-MB-468, BT-549), MDA-MB-361 and HEK293T (human embryonic kidney)) cell lines were obtained from the American Type Culture Collection (ATCC, Manassas, VA, USA). MDA-MB-157, MDA-MB-231, and BT-549 cells are characterized as triple-negative/basal-B mammary carcinoma, while the MDA-MB-468 cells are characterized as triple-negative/basal-A mammary carcinoma. MDA-MB-361 cells are characterized as ER-positive/Progesterone receptor (PgR) negative, luminal mammary carcinoma. Liquid nitrogen stocks were made upon receipt and maintained until the start of each study. MCF-7 cells, characterized as ER-positive/PgR-positive luminal mammary carcinoma, were obtained from frozen stocks routinely used in previous experiments [34]. The ER-positive/PgR-positive ZR-75-1 human epithelial mammary ductal carcinoma cells were a generous gift of Dr. Brian Rowan (Tulane University, New Orleans, LA, USA). Cells were used for no more than six months after being thawed with periodic recording of morphology and doubling times to ensure maintenance of phenotype. Cells were maintained at 37°, 5% CO_2 _in 10% (D)MEM (Invitrogen, Carlsbad, CA, USA) supplemented with 10% fetal bovine serum (Hyclone, Salt Lake City, UT, USA) and 1% penicillin/streptomycin (Invitrogen). Panobinostat was generously provided by Novartis Pharmaceutical Inc. (East Hanover, NJ, USA). Panobinostat was dissolved in dimethyl sulfoxide (DMSO) (Invitrogen) as a 1 mM stock solution and kept at -20°C. The drug was diluted in culture media and used at various concentrations as indicated.

### Histone acetylation

TNBC cells were plated at 70% confluency in 10% (D)MEM and allowed to attach overnight. Cells were treated with panobinostat (100 nM, 200 nM) or vehicle for 18 hours, then fixed, permeabilized and stained with acetyl-histone H3 (Lys9) antibody/Alexa Fluor^® ^488 Conjugate or acetyl-histone H4 (Lys8) antibody/Alexa Fluor^® ^488 Conjugate (Cell Signaling Technology, Danvers, MA, USA), followed by rhodamine phalloidin and DAPI (4',6-diamidino-2-phenylindole) counterstain (Molecular Probes, Carlsbad, CA, USA) according to the manufacturer's instructions. Cells were dually analyzed by BD LSR II flow cytometer and BD Pathway 855 bioimaging confocal system and images merged using BD Attovision™ Software (BD Biosciences, San Jose, CA, USA). Data are represented as mean fluorescence intensity (mean ± SEM) of two independent experiments with internal triplicates.

### MTT cell proliferation assay

Proliferation was measured by MTT (3-(4, 5-dimethylthiazol-2-yl)-2,5-diphenyltetrazolium bromide) Cell Proliferation Assay, according to the manufacturer's protocol (ATCC). Briefly, cells were plated in 96-well flat bottom plates at a density of 5 × 10^3 ^per 100 μl in 10% (D)MEM, allowed to attach overnight, and then treated with panobinostat (50, 100, 200 nM) or vehicle for 24 hours. MTT reagent (10 μl) was added to each well (final concentration 0.5 mg/ml) and the plate incubated at 37°C. After four hours, 100 μl of solubilization solution (10% SDS) was added to each well and the plate incubated for two hours. A matched control cell standard curve using sequentially increased cell numbers was included on the plate for each corresponding cell line to determine growth inhibition. The absorbance was read at 570 nm on a *Synergy*™ 4 Multi-Mode Microplate Reader and analyzed with Gen5™ Data Analysis Software (BioTek, Winooski, VT, USA). Data are represented as mean percent vehicle treated cell proliferation ± SEM of triplicate experiments with internal triplicates.

### Trypan blue viability assay

Cells were plated in 96-well plates at a density of 5 × 10^3 ^per 100 μl in 10% (D)MEM and allowed to adhere overnight. Cells were treated with vehicle or panobinostat (50, 100, 200 nM) for 24 hours and harvested by trypsinization. Cells were then stained with a trypan blue solution (0.04% w/v, Invitrogen), and counted on a Cellometer Vision automated cell counter (Nexcelom Bioscience, Lawrence, MA, USA) according to the manufacturer's protocol. Cell viabilities are represented as mean percent relative to matched, vehicle-treated cells ± SEM of triplicate experiments with internal triplicates.

### Apoptosis

Analysis of apoptosis was carried out using the Cell Death Detection ELISA^PLUS ^according to the manufacture's protocol (Roche Applied Science, Mannheim, Germany). This quantitative DNA fragmentation immunoassay uses monoclonal antibodies directed against histone-complexed DNA. Briefly, cells (10^4 ^cells/well) were plated in 96 well plates overnight and treated for 24 hours with panobinostat (100, 200 nM) or vehicle control. After cell lysis and centrifugation, the cell lysates were tested for histone-complexed DNA fragments. The absorbance was read at 405 nm on a Synergy™ HT Multi-Mode Microplate Reader and analyzed with Gen5™ Data Analysis Software (Bio-Tek). Apoptosis of the treated cells was expressed as mean enrichment factor (treated cells over vehicle controls) ± SEM of duplicate experiments with internal triplicates.

### Cell cycle analysis

For cell cycle analysis, TNBC cells were plated overnight in 10% (D)MEM and treated with 100 nM panobinostat for 24 or 72 hours. Both floating cells and trypsinized adherent cells were collected and combined for analysis. Cells were fixed by dropwise addition into ice cold ethanol and stored at -20° overnight. Cells were then pelleted, washed, and stained for one hour with 50 μg/ml propidium iodide in PBS containing 0.1 mg/ml ribonuclease A and 0.05% Triton X-100 (Sigma, St. Louis, MO, USA). After gating to exclude debris, the DNA content was measured using a LSR-II flow cytometer (BD Biosciences). Data were analyzed with ModFit LT software (Verity Software House, Topsham, ME, USA). Data are represented as percent live cells of two independent experiments.

### Plasmid packaging and stable cell line generation

HEK293T cells were plated at 5.5 × 10^6 ^in a 10 cm dish in 10 ml of 10% (D)MEM and allowed to adhere overnight. The following day, the HEK293t cells were co-transfected with the pLEmiR non-silencing turbo red fluorescent protein (tRFP) vector construct (9 μg) and the trans-lentiviral packaging mix and pLEX™ transfer vector using the TransLenti Viral pLEX packaging system, following the manufacturer's instructions (Thermo Scientific, Waltham, MA, USA). Virus was harvested 48 hours post-transfection and stored at -80°C. TNBC cell lines were plated at 70% confluence in 10 cm dishes with 10 ml of 10% (D)MEM and allowed to adhere overnight. The following day, cells were transduced with virus containing the pLEmiR tRFP vector (1:10 dilution) in serum-free media following the manufacturer's protocol. After four hours, the transduction media was removed and replaced with 10% (D)MEM. After 24 hours, cells were treated with puromycin (Invitrogen) to select for vector expression. The resultant stable transfectants were designated as MDA-MB-231-tRFP and BT-549-tRFP.

### Animal xenograft studies

Xenograft tumor studies were conducted as previously described [34]. In short, CB-17/SCID female mice (four to six weeks old) were obtained from Charles River Laboratories (Wilmington, MA, USA). The animals were allowed a period of adaptation in a sterile and pathogen-free environment with food and water *ad libitum*. MDA-MB-231-tRFP and BT-549-tRFP cells were harvested in the exponential growth phase using a PBS/ethylenediaminetetraacetic acid (EDTA) solution and washed. Viable cells (5 × 10^6^) in 50 μl of sterile PBS suspension were mixed with 100 μl reduced growth factor Matrigel (BD Biosciences) and injected bilaterally into the inguinal mammary fat pad. On day three post cell injection, mice were randomized into treatment groups of five mice each: (vehicle control or 10 mg/kg panobinostat). Beginning on day 14 post cell injection, animals received intraperitoneal (i.p.) injections of the corresponding drug treatment on a five-day on and two-day off schedule for 28 days [18]. Tumor size was measured with a digital caliper and calculated using the formula 4/3πLS^2 ^(L = larger radius, S = smaller radius). At necropsy, animals were euthanized by cervical dislocation following CO_2 _exposure. Tumors, livers, lungs, and brains were removed and snap frozen or fixed in 10% formalin for future analysis. All procedures involving animals were conducted in compliance with State and Federal laws, the U.S. Department of Health and Human Services, and guidelines established by Tulane University Animal Care and Use Committee. The facilities and laboratory animals programs of Tulane University are accredited by the Association for the Assessment and Accreditation of Laboratory Animal Care.

### Human breast cancer quantitative reverse transcription real-time PCR array

Human Breast Cancer and Estrogen Receptor Signaling RT^2 ^Profiler™ PCR Arrays (PAHS-005) were obtained from SABiosciences (Frederick, MD, USA). MDA-MB-231, MCF-7, and MDA-MB-468 cells were plated in 10% (D)MEM at 70% confluency and treated with 100 nM panobinostat or vehicle for 24 hours. Cells were harvested by trypsinization and total RNA was isolated using the RNeasy kit, according to the manufacturer's instructions (Qiagen, Valencia, CA, USA). The quantity and quality of the RNA were determined by absorbance at 260 and 280 nm using the NanoDrop ND-1000 (NanoDrop, Wilmington, DE, USA). Total RNA (1.5 μg) was reverse-transcribed using the RT^2 ^First Strand cDNA synthesis kit, following the manufacturer's protocol (SABiosciences) and then assayed via an optimized, quantitative RT real-time PCR (qPCR) array to assess panobinostat-associated changes in the expression of 84 genes related to breast cancer regulation and estrogen receptor-dependent signal transduction, according to the manufacturer's protocol. Biological triplicates were run for each sample.

### CDH1 flow cytometry and immunofluorescent imaging

MDA-MB-231 cells were plated at 70% confluency in 10% (D)MEM and allowed to attach overnight. Cells were then treated with panobinostat (100 nM) or vehicle for 24 hours. Cells were harvested by gentle pipetting (PBS with 5% fetal bovine serum), fixed, and stained with Alexa Fluor^® ^488-conjugated CDH1 (E-cadherin) antibody (BD Biosciences). The expression of CDH1 protein was determined by flow cytometry on a BD LSRII instrument. Data are represented as mean percent E-cadherin positive cells ± SEM of duplicate experiments with internal triplicates. Paired cells were seeded on BD Falcon 96-well black imaging plates. Staining is represented by the following colors: Gree*n *= CDH1, Red = phaloidin, Blue = DAPI nuclear stain. Confocal immunofluorescent images were captured on the BD Pathway 855 Bioimaging system and merged using BD Attovision™ software (BD Biosciences).

### ELISA for CDH1

MDA-MB-231 cells (10^5 ^cells/well) were plated overnight in six-well plates and then treated for 24 hours with panobinostat (100 nM) or vehicle control. Five volumes of ice-cold lysis buffer (20 mM Tris-HCl, pH 7.5/150 mM NaCl/1 mM EDTA/1 mM ethylene glycol-bis[β-aminoethyl ether]-N, N, N', N'-tetraacetic acid (EGTA)/1% Tween 20) supplemented with protease inhibitor tablets (Roche Diagnostics, Indianapolis, IN, USA) were added to each well. Cell lysates were mechanically dissociated and centrifuged (10,000 × *g *for 15 minutes at 4°C), and then diluted 1:1 with calibrator diluent. CDH1 levels were then determined by human CDH1 ELISA according to the manufacturer's instructions (R&D Systems, Minneapolis, MN, USA). The absorbance was read at 450 nm on a Synergy™ HT Multi-Mode Microplate Reader (Bio-Tek). Data are represented as mean pg/ml of CDH1 ± SEM of duplicate experiments with internal triplicates.

### Immunohistochemical staining

Representative sections of tumor with adjacent tissues were fixed in 10% neutral buffered formalin for 24 to 36 hours. Paraffin-embedded sections were prepared at 4 μm thickness followed by standard H & E staining. Additional sections were manually deparaffinized in xylene, rehydrated in a series of graded ethanol solutions, boiled in10 mM sodium citrate buffer (pH 6.0) for ten minutes, then cooled for 20 minutes for antigen retrieval. Sections were blocked for 30 minutes with 10% normal goat serum (Invitrogen), incubated overnight in a 4° humidified chamber with rabbit anti-E-cadherin (Abcam, Cambridge, UK) at 1:30 dilution, followed by one hour incubation with Alexa Fluor^® ^488 goat anti-rabbit secondary (Invitrogen). Fluorescent images were captured on a Nikon TE2000 inverted microscope with IPLab software (BD Biosciences, Rockville, MD, USA).

### Statistical analyses

Statistical analyses were carried out with GraphPad Prism software (Graph-Pad Software, Inc., San Diego, CA, USA). Studies involving more than two groups were analyzed by one-way analysis of variance (ANOVA) followed by Tukey's post-hoc multiple comparison tests. All others were subjected to unpaired Student's t-test, with *P *< 0.05 considered statistically significant.

## Results

### Panobinostat induces histone acetylation

**Figure 1:**
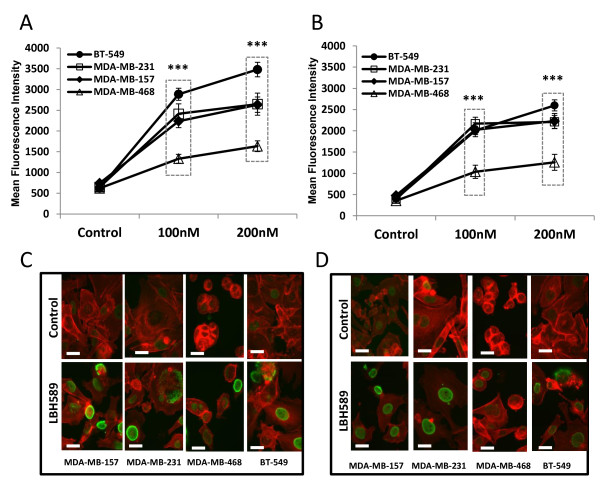
**Panobinostat increases histone H3 (Lys9) and H4 (Lys8) acetylation in TNBC cell lines**. Cells were treated with panobinostat (100, 200 nM) or vehicle (DMSO) for 18 hours, fixed, permeabilized and stained for acetyl-histones **(A) **H3 (Lys9) or **(B) **H4 (Lys8) and subjected to flow cytometry. Data are presented as mean fluorescence intensity (mean ± SEM) of two independent experiments, (***, *P *< 0.001). **(C-D) **Confocal images of TNBC cell lines treated with panobinostat (100 nM) or vehicle for 18 hours, fixed, permeabilized and stained red (rhodamine phalloidin) for actin filaments and green (Alexa Fluor^® ^488) for acetyl-histones **(C) **H3 (Lys9) or **(D) **H4 (Lys8). Original magnification was 400× with scale bars at 20 microns. DMSO, dimethyl sulfoxide; SEM, standard error of the mean; TNBC, triple-negative breast cancer.

To verify the effects of panobinostat as a relevant histone deacetylase inhibitor, four TNBC cell lines, MDA-MB157, MDA-MB-231, MDA-MB-468, and BT-549, were treated with increasing concentrations of the drug (100 to 200 nM) and assayed after 18 hours by flow cytometry for antibodies to acetylated histones H3 and H4. Panobinostat induced hyper-acetylation of histones H3 (Lys9) and H4 (Lys8) in all four tested TNBC cell lines, as seen in Figures 1A and 1B, respectively (***, *P *< 0.001). MDA-MB-468 cells were the least responsive to panobinostat with a 2.1-fold change compared to vehicle treated cells. Additionally, three-color confocal immunofluorescence imaging was conducted to visually confirm the increased accumulation of acetylated histones H3 (Figure 1C) and H4 (Figure 1D) in the panobinostat-treated cells.

### Panobinostat cytotoxicity in TNBC cell lines

**Figure 2:**
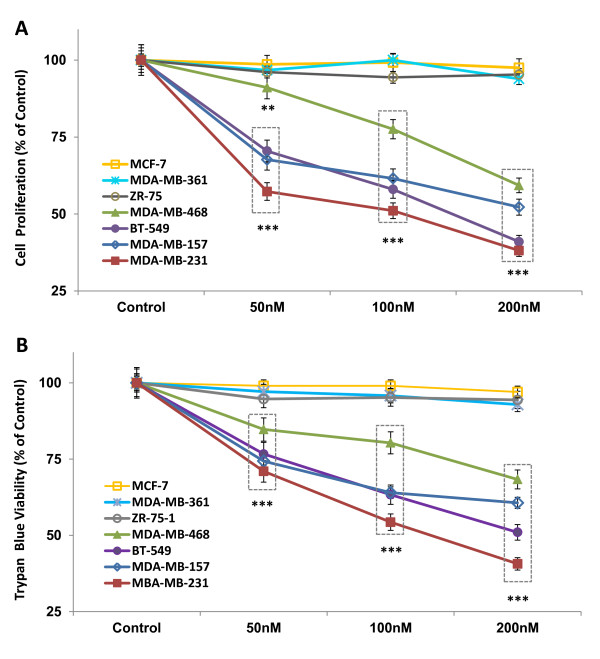
**Panobinostat decreases TNBC cell proliferation and viability**. Cells from four TNBC cell lines (MDA-MB-157, MDA-MB-231, MDA-MB-468, BT549) and three ER-positive cell lines (MCF-7, MDA-MB-361, ZR-75) were treated with panobinostat (50, 100, 200 nM) or vehicle (DMSO) for 24 hours and assayed by **(A) **MTT proliferation and **(B) **trypan blue exclusion assays. Data are represented as percent control (mean ± SEM) of three independent experiments, (**, *P *< 0.01; ***, *P *< 0.001). DMSO, dimethyl sulfoxide; ER, estrogen receptor; MTT, 3-(4,5-dimethylthiazol-2-yl)-2,5-diphenyltetrazolium bromide; SEM, standard error of the mean; TNBC, triple-negative breast cancer.

To determine the effect of panobinostat on cell proliferation and survival *in vitro*, three ER-positive and four TNBC cell lines were treated with increasing doses (50, 100, 200 nM) of the drug for 24 hours. Panobinostat induced a significant dose-dependent decrease in proliferation in all four tested TNBC cell lines, as assayed by MTT metabolism (Figure 2A). At 200 nM, all TNBC cells had a greater than 40% reduction in proliferation compared to vehicle treated cells (*P *< 0.001). In contrast, the growth of ER-positive cell lines (MCF-7, ZR-75-1, and MDA-MB-361) was not significantly affected by panobinostat. In order to confirm the accuracy of the MTT assay, trypan blue assays were also conducted as a measure of membrane integrity (Figure 2B). Again, cell viability was significantly decreased in the TNBC cell lines at all doses compared to vehicle controls, with a greater than 25% decrease in cell viability observed at 200 nM in all the TNBC cell lines (*P *< 0.001). As with the MTT assay, panobinostat treatment did not affect ER-positive cell viability as measured by trypan blue.

The effects of panobinostat on cell cycle progression were analyzed by propidium iodide flow cytometry at 24 and 72 hours. Panobinostat (100 nM) induced G2/M cell cycle arrest, as evidenced by accumulation of cells in G2/M, with a concurrent decrease in S phase peaks in all four tested TNBC cell lines (Table 1). Treatment also induced a time-dependent increase in sub-G/debris fraction in all four TNBC cell lines (data not shown).

**Table 1:** Effect of 100 nM panobinostat on cell cycle percentage of TNBC cells.

	Panobinostat		G0/G1	S	G2/M
MDA-MB-157	-	24 h	61.72	21.68	16.59
	+	24 h	64.3	10.83	24.87
	-	72 h	52.21	25.53	22.26
	+	72 h	54.14	9.48	36.37

MDA-MB-231	-	24 h	86.74	6.03	7.23
	+	24 h	33.04	0.5	66.46
	-	72 h	57.05	24.7	18.25
	+	72 h	37.75	2.33	59.92

MDA-MB-468	-	24 h	54.07	30.29	15.64
	+	24 h	51.55	17.01	31.44
	-	72 h	53.45	34.81	11.74
	+	72 h	52.67	16.78	30.55

BT-549	-	24 h	37.66	26.57	35.77
	+	24 h	39.76	8.61	51.63
	-	72 h	36.42	27.08	36.49
	+	72 h	42.93	3.95	53.13

Data (percent gated cells) representative of two independent experiments. TNBC, triple-negative breast cancer.

**Figure 3:**
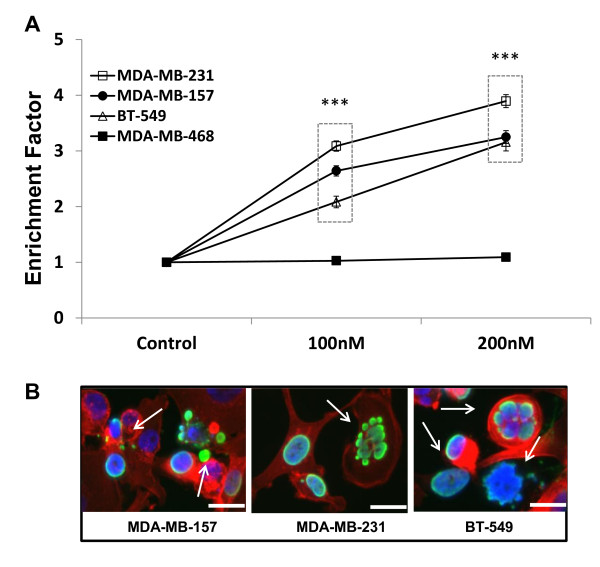
**Panobinostat induces apoptosis in TNBC cells**. **(A) **TNBC cells treated with panobinostat (100, 200 nM) or vehicle (DMSO) for 24 hours were assayed by DNA fragmentation (Cell Death ELISA) assay to assess changes in apoptosis. Data are presented as enrichment (mean ± SEM) versus control of two independent experiments (***, *P *< 0.001). **(B) **Representative confocal images show the presence of apoptotic bodies (arrows) in panobinostat treated MDA-MB-157, MDA-MB-231, and BT-549 cells at 18 hours. Cells stained red (rhodamine phalloidin) for actin filaments, green (Alexa Fluor^® ^488) for acetyl-histone H3 (Lys9), and blue for DAPI nuclear stain. Original magnification is 400× with scale bars at 20 microns. DAPI, 4',6-diamidino-2-phenylindole; DMSO, dimethyl sulfoxide; SEM, standard error of the mean; TNBC, triple-negative breast cancer.

Panobinostat-induced apoptosis, as measured by DNA fragmentation, was assessed at 24 hours in the TNBC cell lines. A clear induction of apoptosis was apparent at 100 nM and 200 nM concentrations in three of the four tested TNBC cell lines (*P *< 0.001), with a mean increase of 304 ± 0.78% at 200 nM (Figure 3A). Enrichment was not significant in the MDA-MB-468 cell line at this time point. Visual evidence of panobinostat-induced apoptosis (arrows) is presented in the panel of confocal immunofluorescence images shown in Figures 3B.

### Panobinostat targets tumor growth *in vivo*

**Figure 4:**
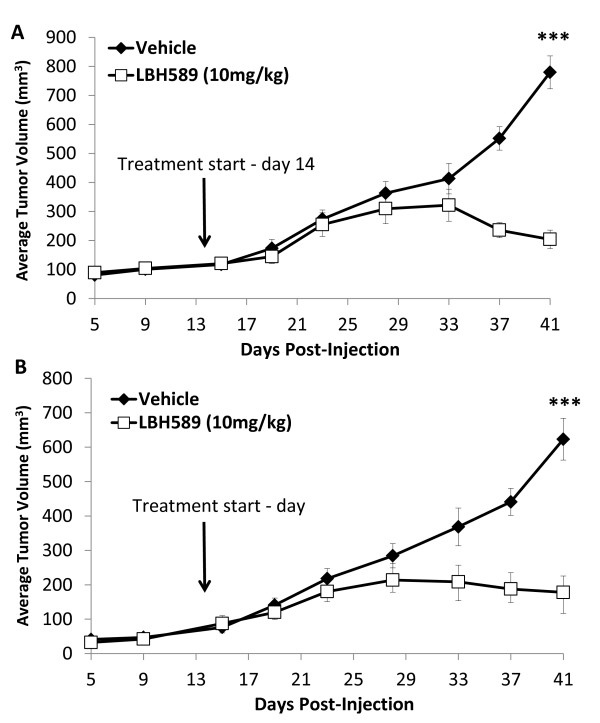
**Effect of panobinostat on tumor growth in MDA-MB-231 and BT549 xenograft models**. Female, CB-17/SCID mice (*n *= 5/group) were injected with **(A) **MDA-MB-231-tRFP or **(B) **BT-549-tRFP cells (5 × 10^6 ^cells/injection) bilaterally into the inguinal mammary fat pad. On day 14, mice were treated intraperitoneally (i.p.) with panobinostat (10 mg/kg) or vehicle (1:20 DMSO in normal saline) five days/week for 28 days. Data points represent average tumor volume ± SEM, (***, *P *< 0.001). DMSO, dimethyl sulfoxide; SEM, standard error of the mean; tRFP, turbo red fluorescent protein.

To determine if the anti-cancer effects of panobinostat observed *in vitro *translated to decreased tumorigenesis *in vivo*, immunocompromised female mice were orthotopically inoculated with MDA-MB-231 (Figure 4A) or BT-549 (Figure 4B) cells (5 × 10^6 ^cells/site, bilaterally) and treated with panobinostat or vehicle control. Treatment with panobinostat (10 mg/kg/day, five days/week) resulted in significant decreases in tumor volume with three- to four-fold (BT549 and MDA-MB-231, respectively) inhibition of tumor volumes compared to controls by day 41 (28 days post treatment, *P *< 0.001). There was no overt toxicity, as measured by weight loss, noted at this dose and treatment schedule.

### Panobinostat regulates breast cancer genes and estrogen signaling pathways

To reveal possible molecular mechanisms and signaling pathways involved in TNBC cell response to panobinostat, MDA-MB-231 cells were treated for 24 hours and analyzed with the Human Breast Cancer and Estrogen Receptor Signaling RT^2 ^Profiler™ PCR Array (SABiosciences). As shown in Additional file 1, thirty-five of the eighty-four representative genes were significantly altered at least two-fold (*P *< 0.05). Specifically, expression of twenty-four genes was up-regulated while expression of eleven genes was suppressed. Of particular interest was the 31-fold increase in the documented epithelial cell marker/tumor suppressor, CDH1 [35]. Also noted were decreases in the proliferation marker MKI67 and upregulation of the tight-junction protein, claudin-7.

**Figure 5:**
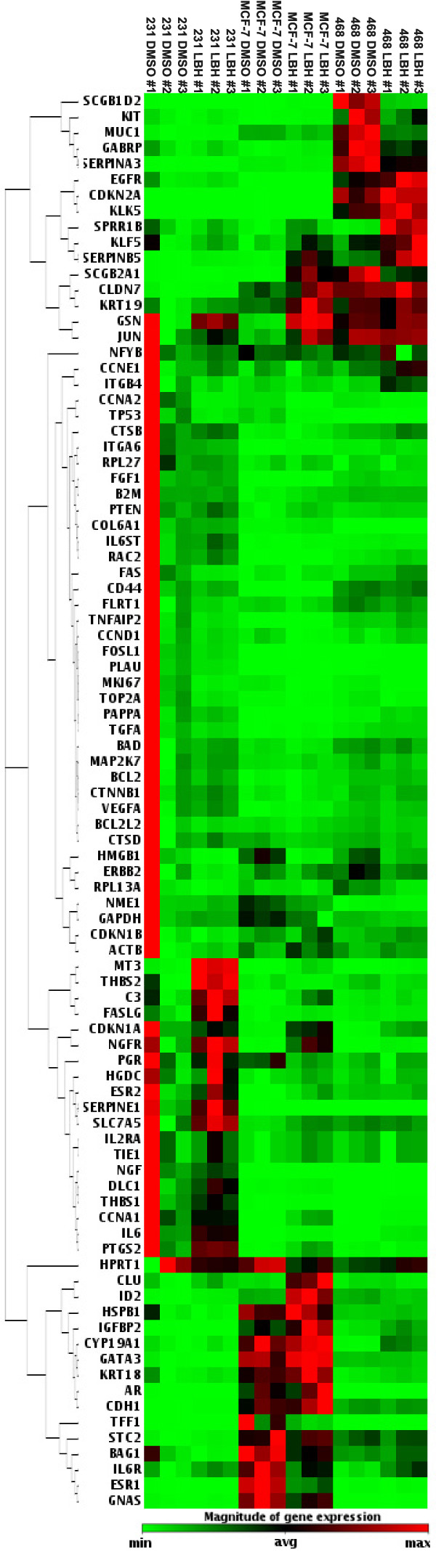
**Heat map of panobinostat -induced gene expression changes in MDA-MB-231, MDA-MB-468, and MCF-7 cells**. MDA-MB-231, MDA-MB-468, or MCF-7 cells were treated for 24 hours with panobinostat (100 nM) or vehicle (DMSO) and then assayed via the Human Breast Cancer and Estrogen Receptor Signaling RT^2 ^Profiler™ PCR Array. Red signifies up-regulation and green signifies down-regulation by panobinostat compared to MDA-MB-231 vehicle treated controls. Data are representative of three independent experiments. Genes regulated at least 2-fold are also summarized in Additional file 1 (MDA-MB-231), 2 (MDA-MB-468) and 3 (MCF-7). DMSO, dimethyl sulfoxide.

To further investigate whether the panobinostat-induced changes discussed above were specific to the basal-B subtype, MDA-MB-468 (basal-A) and MCF-7 (luminal) cell lines were also tested by Human Breast Cancer and Estrogen Receptor Signaling RT^2 ^Profiler™ PCR Array following 24 hours of panobinostat treatment. The representative heat map illustrates the changes in gene expression of all three cell lines following panobinostat treatment as compared to MDA-MB-231 vehicle treated cells (Figure 5). Additional file 2 shows the twenty-four significantly altered genes in the MDA-MB-468 cells following panobinostat treatment (*P *< 0.05), with nineteen genes up-regulated and five genes down-regulated. Of the up-regulated genes, many are known to be involved in the promotion of cell proliferation, survival, and tumor progression (*CCNA1, CCNE1, FOSL1, ITGB4, PAPPA, RAC2, SERPINB5*), while only three tumor suppressive genes (*CDKN1A, SPRR1B, THBS1*) were increased by panobinostat in the MDA-MB-468 cell system. Additional file 3 shows the thirty-four genes significantly altered by panobinostat in MCF-7 cells (*P *< 0.05). Of these altered genes, twenty-four were up-regulated while ten genes were down-regulated. Again, many of the up-regulated genes have known roles in cell proliferation, survival, and tumorigenesis (*CCNA1, FGF1, ITGA6, KLF5, SERPINE1, SLC7A5*) in the MCF-7 cells. Additionally, the well known metastasis suppressor NME1 was decreased by panobinostat in these cells. Overall, these array data reveal a profile consistent with a less aggressive, and more favorable, prognostic profile for MDA-MB-231 cells treated with LHB589, while the less biologically sensitive MDA-MB-468 and MCF-7 cell lines display a less clear cut picture for panobinostat-induced gene expression in cells of the basal-A and luminal subtypes.

### Panobinostat induces changes in morphology and CDH-1 expression of MDA-MB-231 cells consistent with reversal of EMT

**Figure 6:**
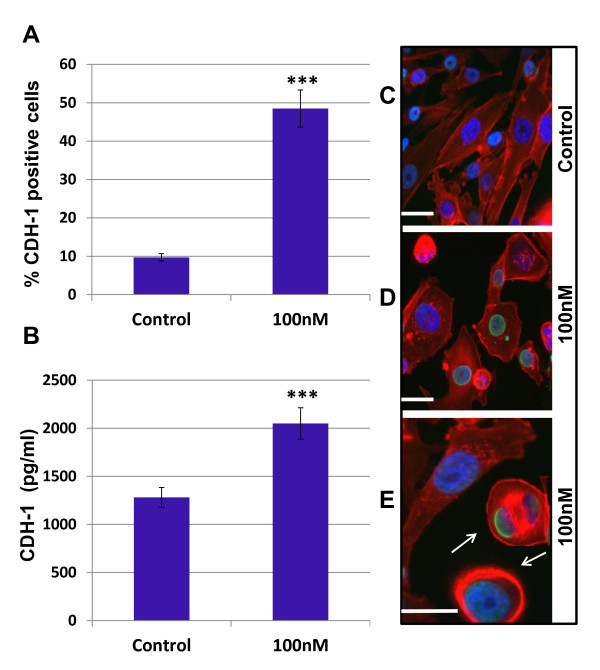
**Panobinostat up-regulates CDH1 expression and initiates EMT reversal in MDA-MB-231 Cells**. MDA-MB-231 cells were plated overnight and treated with panobinostat (100 nM) or vehicle (DMSO) for 24 hours. The expression of CDH1 was examined by **(A) **flow cytometry and **(B) **ELISA. Data are represented as mean ± SEM of two independent experiments, (***, *P *< 0.001). **(C-E) **MDA-MB-231 morphology changes were assessed in vehicle- and panobinostat- (100 n M) treated cells with three-color fluorescence staining on a BD Pathway 855 Bioimager. **(C) **Control and **(D-E) **Panobinostat treated cells were stained red (rhodamine phalloidin) for actin filaments, green (Alexa Fluor^® ^488) for acetyl-histone H3 (Lys9), and blue (DAPI) nuclear counter stain. Partial reversal of EMT in treated cells is indicated by the presence of cuboidal/spherical cells (arrows). **(E) **Two-fold magnification of field with normal untransformed mesenchymal cell and transformed spherical cells. Original magnification is 400× with scale bars at 20 microns. DAPI, 4',6-diamidino-2-phenylindole; DMSO, dimethyl sulfoxide; ELISA, enzyme-linked immunosorbent assay; EMT, epithelial-to-mesenchymal transition; SEM, standard error of the mean.

**Figure 7:**
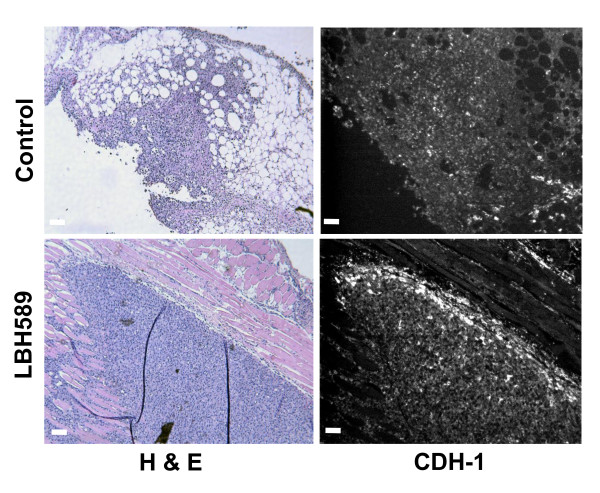
**Panobinostat increases CDH1 expression in MDA-MB-231 primary tumors**. Control and panobinostat treated, formalin-fixed mammary fat pad sections from MDA-MB-231-tRFP injected CB17-SCID mice were stained for H & E (left column) or anti-human CDH1 (1:30; right column) followed by Alexa Fluor^® ^488 secondary antibody. Original magnification was 100× with scale bars at 200 microns. tRFP, turbo red fluorescent protein; SCID, severe combined immunodeficiency.

To assess potential panobinostat-induced changes in morphology and cytoskeletal protein expression in mesenchymal cells, MDA-MB-231 cells were treated with panobinostat (100 nM) for 24 hours and analyzed. Figure 6A shows a significant increase of CDH1-positive cells in panobinostat treated cells compared to control (48.5 ± 2.3% to 9.70 ± 0.569%, respectively; *P *< 0.001). In confirmation, cells were also assayed by ELISA, which showed a 1.6-fold increase in CDH1 protein levels over controls (Figure 6B, *P *< 0.001). These results are consistent with our qPCR array finding of a 31-fold up-regulation of CDH1 expression in MDA-MB-231 cells (Additional file 1). MDA-MB-231 cells also exhibited partial reversal of the mesenchymal phenotype, as evidenced by a shift from spindle shaped cells with visible actin stress fibers to predominantly cuboidal/spherical forms with cortical actin patterns [36-38], following 24-hour treatment with panobinostat (Figure 6C-E). To determine if the *in vitro *up-regulation of CDH1 also occurred *in vivo*, MDA-MB-231 primary tumor sections were stained for CDH1. As can be seen in Figure 7, there is increased CDH1 staining along the periphery of the panobinostat treated tumor.

## Discussion

In recent years, an increasing number of HDACis have been identified, developed and advanced to clinical trials [39,40]. Panobinostat has shown potent activity at low nanomolar concentrations across a wide range of hematologic malignancies and solid tumors in preclinical studies [20-22,41]. Others have demonstrated that HDACi treatment can suppress oncogenes and induce re-expression of previously silenced tumor suppressors and receptors such as the ER [24,42-44]. In addition to its single agent effects, recent studies have demonstrated a role for panobinostat in resensitizing cancer cells to other agents including chemotherapy [45], radiation [46], autophagy inhibitors [47] and endocrine therapies including tamoxifen [48] and letrozole [49]. In consideration of the promising results reported by others, we endeavored to determine whether panobinostat would be effective against a panel of breast cancer cell lines that display common characteristics of the triple-negative subtype.

In this study, we utilized MDA-MB-157, MDA-MB-231, MDA-MB-468, and BT549 cell lines as models of TNBC growth and progression. In confirmation of other preclinical research [20,24,42,44,50,51], we found that panobinostat induced hyperacetylation of histones H3 and H4, decreased proliferation and survival, and induced apoptosis and G2/M cell cycle arrest. The MDA-MB-231 and BT549 lines were chosen as models for our *in vivo *xenograft studies using CB-17/SCID mice. Treatment with panobinostat decreased MDA-MB-231 and BT549 tumor significantly with minimal animal toxicity, providing preclinical data on the effectiveness of panobinostat on TNBC tumorigenesis at a low and well tolerated dose.

The panobinostat-induced effects on cell proliferation and survival appear to be TNBC cell specific as the ER-positive cell lines tested were unaffected at all doses tested (up to 200 nM), contrary to previously published work which reported panobinostat significantly inhibited cell survival and induced cell death in ER-positive and ER-negative breast cancer cell lines though at a different time point [44,47]. We propose that the more aggressive, highly proliferative nature and invasive phenotype of TNBC cells render them particularly susceptible to the effects of panobinostat. Of the four TNBC cell lines tested, the MDA-MB-468 cells were the most resistant to hyper-acetylation and DNA degradation by the drug. This is interesting as this cell line is the most phenotypically different (spherical morphology) and least invasive of the four tested cell lines. The MDA-MB-157, MDA-MB-231, and BT549 lines have been classified as basal-B [52], with the MDA-MB-231 and BT-549 cell lines specifically classified as mesenchymal (stellate), claudin-low, and highly invasive [53-56]. The MDA-MB-157 cells are classified as mesenchymal, claudin-low, and moderately invasive [52]. Clinically, the majority of claudin-low tumors are of the triple-negative subtype and are associated with poor overall prognoses [53]. However, MDA-MB-468 cells have been characterized under the basal-A subtype, as they possess both basal (triple-negative) and luminal (spherical morphology) characteristics and are only minimally invasive [52]. Additionally, super array data comparing panobinostat-induced gene expression changes between panobinostat-sensitive (MDA-MB-231, basal-B) and panobinostat-insensitive (MDA-MB-468, basal-A; MCF-7, luminal) cells revealed several changes specific to the basal-B subtype [See bolded genes in Additional file 1]. These ten genes include known regulators of cell proliferation (*FOSL1, STC2, TGFA, THBS2*) and apoptosis (*FAS, FASLG*), as well as angiogenesis (*TNFAIP2*). Additionally, many of the genes altered by panobinostat specifically in MDA-MB-231 cells have documented roles in cell invasion and metastasis including *CDH1, CLDN7, FOSL1, PLAU, STC2*, and *TGFA*. These data support the role of the selective effects of panobinostat observed on the basal-B cell lines compared to the other subtypes tested.

Interestingly, superarray data identified *CDH1 *as being the most induced gene by panobinostat treatment specifically in MDA-MB-231 cells, as these cells are characterized as mesenchymal, thus lacking significant CDH1 expression. The TNBC subtype is exemplified by its highly aggressive and metastatic nature. A known key step in the process of metastasis is the epithelial-to-mesenchymal transition (EMT). This oncogenic EMT is typified by increased invasion and metastatic dissemination, therapeutic resistance and loss of expression of tumor suppressors such as CDH1 [57,58]. Studies have demonstrated that EMT and the resultant loss of CDH1 expression are crucial steps in tumor progression and correlate with poor clinical outcomes [59-61]. In confirmation of our *in vitro *data on CDH1 up-regulation, we also noted an increase in CDH1 on the periphery of the primary tumor from our MDA-MB-231 xenograft model. Decreased CDH1 expression at the tumor periphery has been linked to increased metastasis-risk and decreased overall patient survival [62]. Induction of CDH1 expression by LHB589 at the invasive edge may therefore be indicative of decreased metastatic potential. Panobinostat-induced re-expression of CDH1, along with other morphological features, indicates the partial reversal of EMT, a target of enormous potential, particularly in the TNBC subtype. This suggests panobinostat as a promising therapeutic option for the more aggressive, TNBC/basal-like breast cancer subtypes.

## Conclusions

Our results illustrate the ability of panobinostat to hyperacetylate histones, inhibit proliferation and survival, and decrease *in vivo *tumorigenesis of TNBC cells. Our *in vitro *data suggest that this cytotoxicity is partially due to cell cycle arrest and apoptosis. Also noted in treated cultures was an apparent partial reversal of the mesenchymal phenotype evidenced by increased CDH1 protein expression and morphology changes in MDA-MB-231 cells. This increased CDH1 was confirmed with measured upregulation of the CDH1 staining at the primary tumor periphery in our xenograft model. Overall, our results affirm the efficacy and demonstrate a potential therapeutic role of panobinostat in targeting aggressive triple-negative breast cancer cell types.

## Abbreviations

ANOVA: analysis of variance; BT-549-tRFP: BT-549 turbo red transfectant; CDH1: cadherin-1, E-cadherin; DAPI: 4',6-diamidino-2-phenylindole; 10% DMEM: Dulbecco's modified Eagle's medium with 10% fetal bovine serum; DMSO: dimethyl sulfoxide; ELISA: enzyme-linked immunosorbent assay; EMT: epithelial-to-mesenchymal transition; ER: estrogen receptor; HDAC: histone deacetylase; HDACi: histone deacetylase inhibitor; H & E: hematoxylin and eosin; Her2/neu: human epidermal growth factor receptor 2; i.p.: intraperitoneal; LBH589: panobinostat; MDA-MB-231-tRFP: MDA-MB-231 turbo red transfectant; MT3: metallothionein 3; MTT: 3-(4,5-dimethylthiazol-2-yl)-2,5-diphenyltetrazolium bromide; PBS: phosphate-buffered saline; PR: progesterone receptor; qPCR: quantitative reverse transcription real-time polymerase chain reaction; SCID: severe combined immunodeficiency; SEM: standard error of the mean; TNBC: triple-negative breast cancer; tRFP: turbo red fluorescent protein.

## Competing interests

The authors declare that they have no competing interests.

## Authors' contributions

CRT drafted the manuscript and conducted histone acetylation, proliferation, viability, apoptosis, cell cycle, flow cytometry, ELISA, and BD pathway analyses, as well as xenograft studies and statistical analyses. LVR generated stable cell lines, conducted the statistical analyses, participated in xenograft experiments, drafting, revising and editing of the manuscript, as well as participated in the concept and design of the studies. HCS performed the qPCR arrays and participated in critical revision of the manuscript. JLD was involved in the xenograft experiments and manuscript revision. FNP conducted the antigen retrieval, staining and visualization of the primary tumor tissues as well as manuscript revision. MEB participated in the concept and design of the study and revising and editing of the manuscript. BMC conceived of the study, participated in the study design and revising and editing of the manuscript. All authors have read and approved of the final manuscript.

## Supplementary Material

Additional file 1 **Panobinostat induced expression changes of breast cancer related genes in MDA-MB-231 cells**. Click here for file

Additional file 2 **Panobinostat induced expression changes of breast cancer related genes in MDA-MB-468 cells**. Click here for file

Additional file 3 **Panobinostat induced expression changes of breast cancer related genes in MCF-7 cells**. Click here for file
