# Supplementary material for: Targeting triple-negative breast cancer cells with the histone deacetylase inhibitor panobinostat
Source: Breast Cancer Res. 2012 May 21;14(3):R79. doi: 10.1186/bcr3192 (PMC3446342; doi:10.1186/bcr3192)
Supplement: Additional file 1 — Panobinostat induced expression changes of breast cancer related genes in MDA-MB-231 cells. [file bcr3192-S1.DOCX]

| **Gene** | **Fold Change** | **p-value** | **Gene** | **Fold Change** | **p-value** |
| --- | --- | --- | --- | --- | --- |
| C3 | 14.2811 | 0.000787 | JUN | 2.0972 | 0.018841 |
| CCNA1 | 2.1187 | 0.000251 | KRT19 | 3.0201 | 0.004639 |
| CCNA2 | -7.9516 | 0.000263 | MKI67 | -5.3952 | 0.003995 |
| CCND1 | -2.3645 | 0.008124 | MT3 | 87.5637 | 0.000006 |
| **CDH1** | 31.5314 | 0.000109 | NGFR | 5.9384 | 0.001183 |
| **CLDN7** | 2.8327 | 0.036800 | **PLAU** | -3.6183 | 0.004041 |
| CLU | 4.833 | 0.021822 | PTGS2 | 3.9591 | 0.000003 |
| DLC1 | 2.4499 | 0.018090 | SCGB2A1 | 2.8009 | 0.012118 |
| ESR2 | 3.1422 | 0.038547 | SERPINB5 | 16.6616 | 0.000233 |
| **FAS** | -2.5629 | 0.000034 | SERPINE1 | 3.6917 | 0.008257 |
| **FASLG** | 9.1408 | 0.010602 | **STC2** | 5.3278 | 0.001767 |
| **FOSL1** | -2.7357 | 0.000378 | TFF1 | 3.6224 | 0.003828 |
| GATA3 | -2.6459 | 0.012088 | **TGFA** | -2.0492 | 0.008134 |
| GSN | 3.2493 | 0.000240 | **THBS2** | 13.9506 | 0.000027 |
| ID2 | 15.6074 | 0.001580 | **TNFAIP2** | -3.0531 | 0.037264 |
| IGFBP2 | 12.9484 | 0.021366 | TOP2A | -3.5808 | 0.010860 |
| IL6 | 2.7785 | 0.000187 | TP53 | -5.7928 | 0.004610 |
| IL6R | 4.7075 | 0.002408 |  |  |  |

**Supplemental Table 1.** **Panobinostat induced expression changes of breast cancer related genes in MDA-MB-231 cells.**

Data (expressed as fold change vs. controls) representative of three independent experiments (p<0.05). Up-regulated genes are in red, down-regulated genes are in blue.
