# Supplementary material for: Targeting triple-negative breast cancer cells with the histone deacetylase inhibitor panobinostat
Source: Breast Cancer Res. 2012 May 21;14(3):R79. doi: 10.1186/bcr3192 (PMC3446342; doi:10.1186/bcr3192)
Supplement: Additional file 2 — Panobinostat induced expression changes of breast cancer related genes in MDA-MB-468 cells. [file bcr3192-S2.DOCX]

| **Gene** | **Fold Change** | **p-value** | **Gene** | **Fold Change** | **p-value** |
| --- | --- | --- | --- | --- | --- |
| CCNA1 | 25.69 | 0.004405 | MT3 | 2.58 | 0.047406 |
| CCNA2 | -2.89 | 0.000510 | MUC1 | -3.26 | 0.004271 |
| CCNE1 | 2.06 | 0.034962 | NGFR | 8.25 | 0.001621 |
| CDKN1A | 4.57 | 0.001671 | PAPPA | 3.46 | 0.002328 |
| COL6A1 | 9.08 | 0.000665 | RAC2 | 2.28 | 0.023474 |
| CYP19A1 | 10.69 | 0.001384 | SCGB1D2 | -10.05 | 0.000329 |
| FOSL1 | 3.88 | 0.009441 | SERPINB5 | 3.29 | 0.011926 |
| IGFBP2 | 3.87 | 0.005641 | SERPINE1 | 9.40 | 0.004054 |
| IL6 | 36.21 | 0.000351 | SPRR1B | 10.61 | 0.000177 |
| IL6R | 4.32 | 0.012499 | TFF1 | 7.29 | 0.002060 |
| ITGB4 | 3.15 | 0.001689 | THBS1 | 2.08 | 0.001811 |
| MKI67 | -2.66 | 0.005104 | TOP2A | -2.85 | 0.047559 |

**Supplemental Table 2.** **Panobinostat induced expression changes of breast cancer related genes in MDA-MB-468 cells.**

Data (expressed as fold change vs. controls) representative of three independent experiments (p<0.05). Up-regulated genes are in red, down-regulated genes are in blue.
