# Supplementary material for: Targeting triple-negative breast cancer cells with the histone deacetylase inhibitor panobinostat
Source: Breast Cancer Res. 2012 May 21;14(3):R79. doi: 10.1186/bcr3192 (PMC3446342; doi:10.1186/bcr3192)
Supplement: Additional file 3 — Panobinostat induced expression changes of breast cancer related genes in MCF-7 cells. [file bcr3192-S3.DOCX]

| **Gene** | **Fold Change** | **p-value** | **Gene** | **Fold Change** | **p-value** |
| --- | --- | --- | --- | --- | --- |
| C3 | 221.67 | 0.036263 | JUN | 3.90 | 0.028618 |
| CCNA1 | 133.37 | 0.011505 | KLF5 | 2.93 | 0.043037 |
| CCNA2 | -11.71 | 0.000044 | KLK5 | 6.48 | 0.015973 |
| CCND1 | -5.47 | 0.041624 | KRT19 | 2.22 | 0.027859 |
| CDKN1A | 7.16 | 0.000022 | MKI67 | -8.09 | 0.001231 |
| CLU | 8.79 | 0.000165 | MT3 | 13.03 | 0.035126 |
| COL6A1 | 2.91 | 0.002925 | NGFR | 87.64 | 0.012465 |
| DLC1 | 2.46 | 0.021622 | NME1 | -2.19 | 0.006751 |
| ESR1 | -4.13 | 0.000053 | PGR | -2.02 | 0.035412 |
| ESR2 | 3.18 | 0.038440 | PTGS2 | 4.76 | 0.024590 |
| FGF1 | 2.63 | 0.013574 | SCGB1D2 | 7.01 | 0.019844 |
| GSN | 3.40 | 0.000614 | SCGB2A1 | 106.52 | 0.005723 |
| HMGB1 | -4.49 | 0.003741 | SERPINB5 | 32.51 | 0.005101 |
| ID2 | 4.90 | 0.005877 | SERPINE1 | 43.76 | 0.001627 |
| IL6 | 26.13 | 0.000382 | SLC7A5 | 2.70 | 0.005534 |
| IL6R | -2.44 | 0.012947 | TOP2A | -2.75 | 0.008773 |
| ITGA6 | 4.07 | 0.006897 | TP53 | -12.06 | 0.006725 |

**Supplemental Table 3.** **Panobinostat induced expression changes of breast cancer related genes in MCF-7 cells.**

Data (expressed as fold change vs. controls) representative of three independent experiments (p<0.05). Up-regulated genes are in red, down-regulated genes are in blue.
